# Supplementary material for: Modulating DNA Polα Enhances Cell Reprogramming Across Species
Source: bioRxiv. 2024 Sep 20:2024.09.19.613993. Preprint. [Version 1] doi: 10.1101/2024.09.19.613993 (PMC11429986; doi:10.1101/2024.09.19.613993)
Supplement: 1 [file NIHPP2024.09.19.613993V1-supplement-1.pdf]

bioRxiv preprint doi: https://doi.org/10.1101/2024.09.19.613993; this version posted September 20, 2024. The copyright holder for this preprint (which was not certified by peer review) is the author/funder, who has granted bioRxiv a license to display the preprint in perpetuity. It is made available under aCC-BY 4.0 International license.

Supplemental Information

Materials and Methods

***Drosophila* strains and husbandry.** Fly strains were raised on standard Bloomington media. All flies were raised at 25°C unless noted otherwise. The following fly strains were used: *polα50* P-element insertion (BL-27205) (*51*), *nos-Gal4 (with VP16)* on the 2nd chromosome (*112*), *nos-Gal4* (*without VP16* or *ΔVP16*) on the 2nd chromosome [(from Yukiko Yamashita, Whitehead Institute, USA) and used in (*113*)], *Delta-nuclear lacZ* reporter on the 3rd chromosome (Dr. Allan Spradling, Carnegie Institute, USA). The *UAS-grim* flies (*78*) (from Erika Matunis, Johns Hopkins School of Medicine, USA) were crossed with *nanos-Gal4ΔVP16; tubulin-Gal80^{ts}* for genetic ablation experiments to induce dedifferentiation (see below).

All experiments using the *pola50$^{+/-}$* were outcrossing the *pola50*/Balancer stock to a wild-type stock (*y,w*) to have the *pola50* P-element insertion allele over a wild-type chromosome. To avoid any potential effects brought by outcrossing, control flies were from outcrossing two wild-type strains: Oregon-R and *y,w*.

***C. elegans* strain maintenance.** *C. elegans* were maintained on nematode growth medium agar plates using Escherichia coli OP50 as a food source and cultured according to standard methods (*114*). The following strains were used in this study: N2, VC4505 [*pola-1(gk5576)III/+* heterozygotes], CB1370 [*daf-2(e1370) III*], GC1413 rrf-1[*pk1417; naSi2 (Pmex5::H2B::mCherry::nos-2 3'UTR); teIs113 (Ppie-1::GFP::H2B::zif-1 3'UTR)*] (from Jane Hubbard, New York University, USA).

bioRxiv preprint doi: https://doi.org/10.1101/2024.09.19.613993; this version posted September 20, 2024. The copyright holder for this preprint (which was not certified by peer review) is the author/funder, who has granted bioRxiv a license to display the preprint in perpetuity. It is made available under aCC-BY 4.0 International license.

Immunostaining. Immunostaining experiments were performed using standard procedure for *Drosophila* testes (*115*), ovaries (*116*) and midguts (*117*). All secondary antibodies were the Alexa Fluor-conjugated series (1:1,000; Molecular Probes).

For immunostaining of *Drosophila* testes, primary antibodies used were DE-cadherin (10:200; DHSB AB_528120), γ-Tubulin (1:200; Sigma-Aldrich AB_T6557), Stat92E (1:500; from Denise Montell, University of Santa Barbara, CA, USA), VASA (1:500; from Ruth Lehmann, Whitehead Institute, USA), Armadillo (1:100; DSHB N2 7A1), Traffic Jam (1:100, from Mark Van Doren, Johns Hopkins University, USA), anti- H4K20me2/3 (1:400; Abcam ab78517), and anti-H3S10ph (1:2000; Cell Signaling Technology 9701).

For immunostaining of *Drosophila* ovaries, ovaries were fixed in 4% formaldehyde in 0.3% PBST (1x PBS, 0.3% Triton X-100), washed twice for 10 min in 0.3% PBST, and blocked in 5% normal goat serum (NGS, Jackson ImmunoResearch lab, 005-000-121) in 1% PBST (1x PBS, 0.1% Triton X-100) overnight, followed by a two-day primary antibody incubation at 4°C (diluted in 0.3% PBST with 5% NGS). Then, ovaries were washed three times for 20 min in 0.3% PBST and twice for 30 min in 0.3% PSBT with 5% NGS, and incubated with secondary antibodies for 2 hours (diluted in 0.3% PBST with 5% NGS) at room temperature. In the last 30 min of secondary incubation, Hoechst (Thermo Fisher Scientific, 33342) was added. Last, they were washed three times for 20 min in 0.3% PBST before mounting in Vectashield (Vector Laboratories H100010). Primary antibodies include pMAD (1:800; Cell Signaling Technology 9516), Hts (1:20; DSHB, 1B1), α-spectrin (1:50; DSHB, 3A9), Armadillo (1:50; DSHB, N2 7A1), and LaminC (1:100; DSHB, LC28.26).

For Immunostaining of *Drosophila* midgut, fly intestines were dissected in pre-chilled Schneider's media within 30 minutes, followed by fixation with 4% formaldehyde in PBST (1X

bioRxiv preprint doi: https://doi.org/10.1101/2024.09.19.613993; this version posted September 20, 2024. The copyright holder for this preprint (which was not certified by peer review) is the author/funder, who has granted bioRxiv a license to display the preprint in perpetuity. It is made available under aCC-BY 4.0 International license.

PBS+0.1% TritonX-100) for 1 hour at room temperature on a nutator. Wash tissues in PBST for 10 minutes, repeating twice and perform an additional wash for 30 minutes. Block in 5% NGS + 1% BSA (Bovine Serum lbumin) for at least two hours at room temperature or overnight at 4°. Primary antibodies include β-Galactosidase (1:500; Abcam ab9361), H3T3P (1:500; Millipore Sigma 05-746R), diluted in PBST +5% NGS + 1% BSA for overnight incubation at 4°. Wash tissues in PBST for 10 minutes, repeating twice and perform an additional wash for 30 minutes. After secondary antibody incubation with secondary antibodies (diluted in PBST +5% NGS + 1% BSA) for 2 hours at RT. Wash tissues in PBST for 10 minutes, repeating twice and perform an additional wash in PBST for 30 minutes. Remove the last wash and mount the tissue with Fluoromount-G™ Mounting Medium with DAPI (Invitrogen,00-4959-52). The stitched images were then converted into Imaris file format (Imaris 10.1 (RRID:SCR_007370) and the number of both the nLacZ positive cells and DAPI were counted using the built-in spot analysis on Imaris. We then manually checked the cell count to filter out any false positive and false negative signals mis-detected by the software.

Quantification of pMAD immunostaining signals. To measure pMAD signals, z-stacks (sum of slices) were generated at 0.5 μm intervals of individual germarium. Using the draw tool in Fiji, a circle indicating the GSC nuclei based on Hoechst staining was drawn and the mean fluorescence intensity (MFI) was taken. Another circle was drawn in Region 2b and the mean fluorescence intensity was taken as background (BG). Stemness Index was defined as (MFI - BG)/BG to eliminate batch difference. GSCs were identified and quantified, based on the spectrosome morphology and position: round α-spectrin signals at the anterior tip of the germarium and next to cap cells.

bioRxiv preprint doi: https://doi.org/10.1101/2024.09.19.613993; this version posted September 20, 2024. The copyright holder for this preprint (which was not certified by peer review) is the author/funder, who has granted bioRxiv a license to display the preprint in perpetuity. It is made available under aCC-BY 4.0 International license.

Tile scan imaging of the *Drosophila* gut. To examine regeneration ability during gut infection, we conducted tile scan imaging of the entire gut with high spatial resolution. For this, adult *Drosophila* intestines were immunestained and mounted on slides for imaging. All tile scans were performed using a spinning disk confocal microscope equipped with two qCMOS QUEST cameras (Hamamatsu), an X-Light V3 confocal spinning disk system, a high precision Piezo XY stage, and a LDI-laser module. Images were acquired using a 63x Olympus oil object. The VisiView software (BioVision) was used to outline the gut, set the Z-stack, select individual lasers, and acquire images with 2x2 binning. After acquiring individual tile scans, VisiView software was also used to stitch them together, reconstructing the entire gut. The images were processed using Fiji software (to convert them into TIFF file format) and Imaris software for 3D image reconstruction. The number of intestinal stem cells (ISCs) and other cell counts in the gut were quantified using Imaris software (Bitplane).

Bacterial infections. *C. subtsugae* Δ*vioS* (Δ*vioS*) was grown in LB (Invitrogen) media inoculated with a single bacterial colony, taken from solid medium cultures grown from glycerol stocks kept at -80°C, and streaked fresh every week. All bacterial inocula were prepared from overnight liquid cultures, shaking at 30°C for 18 hours. The cultures were then diluted in phosphate buffer saline (PBS) to OD_{600}=100. *Drosophila* adults were starved in empty fly vials for two hours at 29°C, after which they are added to food containing a filter soaked in a 1:1 mixture of 2.5% sucrose and the prepared bacterial suspension or LB media for controls. For each infection tube, 75µL of homogenous bacterial pellet mix (OD_{600}=200) was mixed with an equal volume of 2.5% sucrose solution. In the non-infected control group, 75 µL of LB media

bioRxiv preprint doi: https://doi.org/10.1101/2024.09.19.613993; this version posted September 20, 2024. The copyright holder for this preprint (which was not certified by peer review) is the author/funder, who has granted bioRxiv a license to display the preprint in perpetuity. It is made available under aCC-BY 4.0 International license.

was mixed with an equal volume of 2.5% sucrose solution. Flies were fed the inoculum for 24 hours and then were flipped onto new food or smurf food (normal food with FD&C Blue Dye 1 added for smurf assay). Infection vials with flies were maintained at 29°C, and death was recorded once a day for a number of days to monitor survival over time. For the Smurf assay, in addition to the number of deaths flies exhibiting systematic blue pigmentation were counted. Any flies that died without blue pigmentation were also noted.

Survival and smurf assays post-infection: On the day designated for the infection assay (Day 0), 30 two-day old female flies were sorted for each infection condition, which are placed into empty vials and incubated at 29°C for two hours and subsequently flipped into infection tubes. Dead flies recorded within this window of time are censored as infection-independent deaths.

For survival assay, following a 24-hour infection period, flies were transferred into vials containing filter paper soaked with 200uL 2.5% sucrose solution. The number of deaths was recorded every 24 hours, and flies were flipped into vials with fresh 2.5% sucrose every two days.

For smurf assay, following a 24-hour infection period, flies were transferred into vials containing smurf food. The number of dead flies was recorded and the flies were flipped into vials with fresh smurf food every 24 hours. Notably, the bodies of most deceased flies exhibited systematic blue pigmentation. Any flies that died without blue pigmentation were also noted.

Genetic ablation and regeneration assay in *Drosophila* male germline. GSCs and early-stage germ cells (up to 4-cell spermatogonia) were depleted using genetic manipulation of the pro-

bioRxiv preprint doi: https://doi.org/10.1101/2024.09.19.613993; this version posted September 20, 2024. The copyright holder for this preprint (which was not certified by peer review) is the author/funder, who has granted bioRxiv a license to display the preprint in perpetuity. It is made available under aCC-BY 4.0 International license.

apoptotic gene, *grim*. The *UAS-grim* flies were crossed with *nanos-Gal4ΔVP16; tubulin-Gal80^{ts}* and grown at 18°C to prevent *grim* expression, which is only turned on when shifting flies to 31°C to inactivate Gal80. The *nanos-Gal4ΔVP16; tubulin-Gal80^{ts}>UAS-grim* flies were kept at 31°C for four days, which ablates early-stage germ cells (Abl in Fig. 4H, 4J). After 4-day ablation, flies were recovered at 18°C for another four days, when *grim* expression was inhibited again (Recv in Fig. 4H, 4J). After recovery, all GSCs including dedifferentiated GSC-like cells and *bona fide* GSCs were evaluated by immunostaining using anti-γ-Tubulin as the centrosome marker (*46*) and anti-Stat92E as the stemness marker (*54-57*).

Centrosome orientation assay. During aging or regeneration, both dedifferentiated GSC-like cells and *bona fide* GSCs were evaluated using centrosome orientation as a criterion, as shown previously (*18*) and illustrated (Fig. 4I, purple dot = centrosome). Centrosomes were immunostained using anti-γ-Tubulin(*46*). Centrosome misorientation is defined as neither of the two centrosomes being within the 90° hub–GSC interface (red in Fig. 4I). Centrosomes were scored to be oriented when one of two centrosomes is within the 90° hub–GSC interface (Fig. 4I).

***Drosophila* fertility assays**. *polα50* P-element insertion flies were maintained over a balancer on the 3rd chromosome. The *polα50$^{+/-}$* flies used for fertility assays are from outcrossing the *polα50*/*Balancer* stock with *y,w* flies at 25°C to generate the F1 progeny, where the *polα50* P-element insertion allele is over a wild-type chromosome. As a control, *y,w* flies were crossed to another *wild-type* strain Oregon R flies. The F1 progeny for both *polα50$^{+/-}$* and control were collected on the day they eclosed and aged simultaneously in separate vials.

bioRxiv preprint doi: https://doi.org/10.1101/2024.09.19.613993; this version posted September 20, 2024. The copyright holder for this preprint (which was not certified by peer review) is the author/funder, who has granted bioRxiv a license to display the preprint in perpetuity. It is made available under aCC-BY 4.0 International license.

For male fertility assays, the F1 *polα50*$^{+/-}$ or control males were aged at 25°C for the mentioned period of time with females. Vials were flipped every five days to prevent mixture with F2 progeny. When male flies (*polα50*$^{+/-}$ and *control*$^{+/+}$) reached the desired age, one male was put into a new vial with three virgin *y,w* females. These flies were allowed to mate for five days, and then the parents were tossed away. Only those vials with all four parents (1 male and 3 female) alive after the 5-day mating period were retained for fertility assay. If any of the four parental flies died during the 5-day mating period, those vials were excluded from data collection and analyses. All F2 progeny were counted for 17-18 days after tossing the parent flies. Each mating from one male with three *y,w* females was recorded as one data point.

For female fertility assays, the F1 *polα50*$^{+/-}$ of control females were isolated as virgins and aged at 25°C in the absence of males to prevent mating before the assay. When female flies reached the desired age, one female was put into a new vial with two male *y,w* flies that had eclosed less than 24 hours previously. These flies were allowed to mate for five days, and then the parents were tossed away. Similar to the male fertility assay, only vials in which all parents survived the 5-day mating period were retained for the assay. All F2 progeny were counted for 15 days after tossing the parent flies. Each mating from one female with two *y,w* males was recorded as one data point.

***C. elegans* fertility assays**. Manually selected L4 animals were grown individually on petri dishes seeded with OP50 *E. coli* food. They were then transferred on a new plate every 24 hours). The brood size of each worm was scored by counting the total number of larvae laid on the plates. For each brood size experiment, at least 30 worms were scored for each strain.

bioRxiv preprint doi: https://doi.org/10.1101/2024.09.19.613993; this version posted September 20, 2024. The copyright holder for this preprint (which was not certified by peer review) is the author/funder, who has granted bioRxiv a license to display the preprint in perpetuity. It is made available under aCC-BY 4.0 International license.

For hermaphrodite brood assays, the VC4505 strain is a CRISPR/Cas9 gene deletion of *pola-1*[*gk5576)III*] and maintained as a heterozygous strain. *pola-1(gk5576)/+* heterozygotes were used for experiments (*75*). As a control, the F1 progeny of both *pola-1(gk5576)* and the wild-type strain, N2, were scored in parallel on the same days.

Analysis of *C. elegans* germline GSC/progenitor zone. The strain GC1413 rrf-1(pk1417); naSi2 (Pmex-5::H2B::mCherry::nos-2 3′UTR); teIs113 (Ppie-1::GFP::H2B::zif-1 3′UTR) was used to label all germline nuclei with mCherry (red), while progenitor zone nuclei are doubly marked with GFP and mCherry (yellow) in both wild-type and *pola-1(gk5576)/+* heterozygote backgrounds. Quantification of each region was measured by counting the rows of cells from the distal end.

***Drosophila* lifespan assay.** To assess the lifespan of *polα50*$^{+/-}$ and control flies, we adapted a protocol from (*73*). Groups of 20 newly eclosed flies (10 males and 10 females) were placed in vials with fly food and yeast. The day the flies eclosed was considered Day 0, and flies were kept in a temperature-controlled 25°C incubator for their entire lifespan. Every 2-3 days, flies were flipped onto fresh fly food with dry yeast, and deceased flies were removed and counted. Prior to Day 30, if all the males or all the females in a vial died (leaving all remaining flies as the same sex), then new flies of the missing sex were added, but not counted in the lifespan assay, to maintain the effects of mating in the remaining flies. Deceased flies were counted with respect to sex, but no significant difference was observed for control *versus* experimental males or control *versus* experimental females (data not shown).

bioRxiv preprint doi: https://doi.org/10.1101/2024.09.19.613993; this version posted September 20, 2024. The copyright holder for this preprint (which was not certified by peer review) is the author/funder, who has granted bioRxiv a license to display the preprint in perpetuity. It is made available under aCC-BY 4.0 International license.

***C. elegans* lifespan assays**. All strains were maintained at 20°C. For each strain, gravid adults were bleached to isolate embryos. Embryos were then placed in liquid overnight to obtain a synchronized population of L1 worms that were then plated and allowed to grow to young adults. Strains N2 (n=68), CB1370 (n=33), and VC4505 (n=59), were transferred onto freshly seeded plates and scored by gently tapping with a platinum wire every 2-3 days.

Generation of human induced pluripotent stem cells with PolA1. To generate iPSC lines from human dermal fibroblast cells, the cells before five passages were used for the reprogramming experiments. Prior the CytoTune™-iPS 2.0 Sendai transfection, plate fibroblasts onto 6-well plates at ~40% density with 2×10^5– 3×10^5 cells per well. During the early reprogramming stage, the 10-20 nM PolA1 inhibitor was added to the fibroblast growth medium: FBM™ Basal Medium with FGM™-2 SingleQuots™ supplements (Lonza, CC-3131) until Day 7 and the fibroblast growth medium with PolA1 needs to be replaced daily. After that, the cells were passaged onto the vitronectin-coated dishes with 5×10^4 cells per well and incubated 24 hours in a 37°C incubator with a humidified atmosphere of 5% CO2. Then change the medium to Essential 8™ Medium (Thermo Fisher, A1517001) to maintain the growth of programmed cells. The E8 medium needs to be replaced daily. Until Day 21, the colonies should have grown to an appropriate size for transfer. For the first three passages, manually cut the iPSCs colonies into small pieces and transfer onto a new vitronectin-coated dish. Allow the colonies to attach for 48 hours before replacing the spent medium with fresh E8 medium. When the colonies cover ~80% of the surface area, passage the colonies using 0.5 mM EDTA prepared in Dulbecco’s Phosphate-

bioRxiv preprint doi: https://doi.org/10.1101/2024.09.19.613993; this version posted September 20, 2024. The copyright holder for this preprint (which was not certified by peer review) is the author/funder, who has granted bioRxiv a license to display the preprint in perpetuity. It is made available under aCC-BY 4.0 International license.

Buffered Saline (DPBS) without calcium or magnesium. 10 μM Y-27632 was added to the E8 medium for one day for every passage.

Single-nucleus RNA-seq and data analysis. Colonies of induced human pluripotent stem cells (iPSCs) were picked and cultured in E8 medium until passage 9 at which time point single nuclei were isolated following 10× Genomics Protocol CG000365. One iPSC line with PolA1 inhibitor treatment and one without treatment (control) were used for experiments and data analysis. Single-nucleus RNA-seq (snRNA-seq) libraries were prepared following 10x Genomics product instructions (Catalog# PN-1000283). The final libraries were sequenced by Novogene USA with paired-end 150 bp setting. The raw sequencing results were examined by fastqc v0.12.1 (https://www.bioinformatics.babraham.ac.uk/projects/fastqc/) before processing by cellranger v2.0.2 with GRCh38 as the reference genome. Publicly available single-cell RNA-seq datasets of human iPSCs were downloaded from ArrayExpress (accession number: E-MTAB-6524) (*101*) and Gene Expression Omnibus (accession number: GSE197380) (*102*) and used for comparison. RNA-seq count matrices of all samples were analyzed by Seurat v5.1.0 (*118*) after removing doublets with scDBlFinder v1.14.0 (*119*). The "CCAIntegration" method in Seurat was used to remove batch effects among samples before visualization in UMAP. The newly generated snRNA-seq data of iPSCs with or without PolA1 inhibitor treatment during induction were compared with published iPSCs scRNA-seq data, visualized in the same plot or separate plots.

bioRxiv preprint doi: https://doi.org/10.1101/2024.09.19.613993; this version posted September 20, 2024. The copyright holder for this preprint (which was not certified by peer review) is the author/funder, who has granted bioRxiv a license to display the preprint in perpetuity. It is made available under aCC-BY 4.0 International license.

Supplemental Figures and Figure Legends:

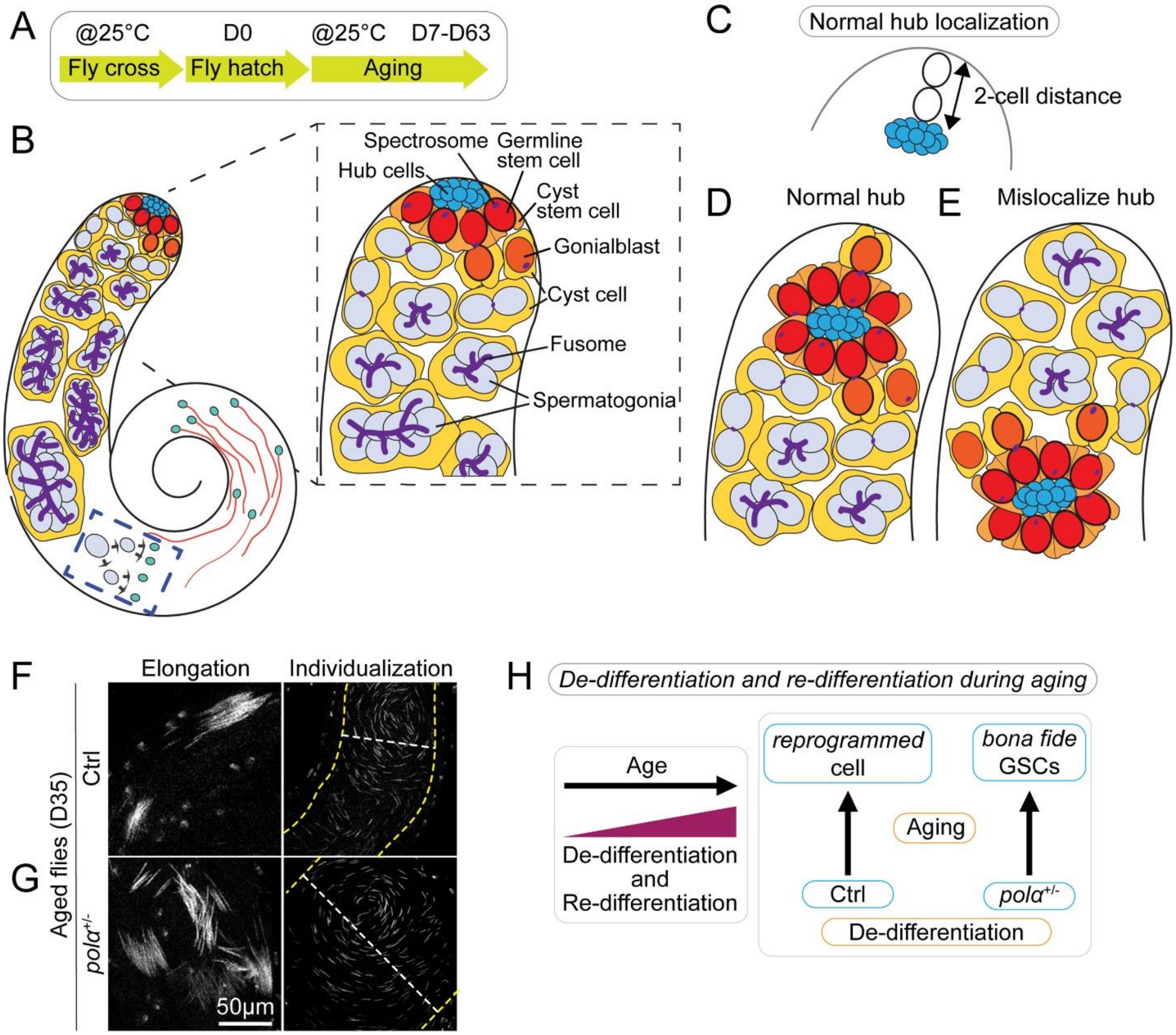

Figure S1: Design and results of the fertility assay in males with reduced Polα levels. (**A**) Regime of aging adult male flies at 25°C after eclosion (D0) up to 63 days (D63, see Materials and Methods). (**B**) A cartoon depicting a *Drosophila* testis and the apical tip showing different cell types and their characteristic cellular features. (**C-D**) A cartoon illustrating the apical tip of the testis with normal niche anatomy (**D**) and the criterion for normal hub localization (**C**). (**E**) A cartoon illustrating abnormal niche anatomy, with the hub structure mislocalized toward the middle of the testis. (**F-G**) DAPI staining of the spermatid elongation and individualization

bioRxiv preprint doi: https://doi.org/10.1101/2024.09.19.613993; this version posted September 20, 2024. The copyright holder for this preprint (which was not certified by peer review) is the author/funder, who has granted bioRxiv a license to display the preprint in perpetuity. It is made available under aCC-BY 4.0 International license.

regions of the control (**F**) and *polα50*$^{+/-}$ (**G**) flies at D35 (35 days after eclosion). Scale bar: 50μm. The yellow dotted lines outline the testis in and white dotted line indicates the width of the basal part of testis. (**H**) A scheme showing the dedifferentiation and redifferentiation processes during aging.

bioRxiv preprint doi: https://doi.org/10.1101/2024.09.19.613993; this version posted September 20, 2024. The copyright holder for this preprint (which was not certified by peer review) is the author/funder, who has granted bioRxiv a license to display the preprint in perpetuity. It is made available under aCC-BY 4.0 International license.

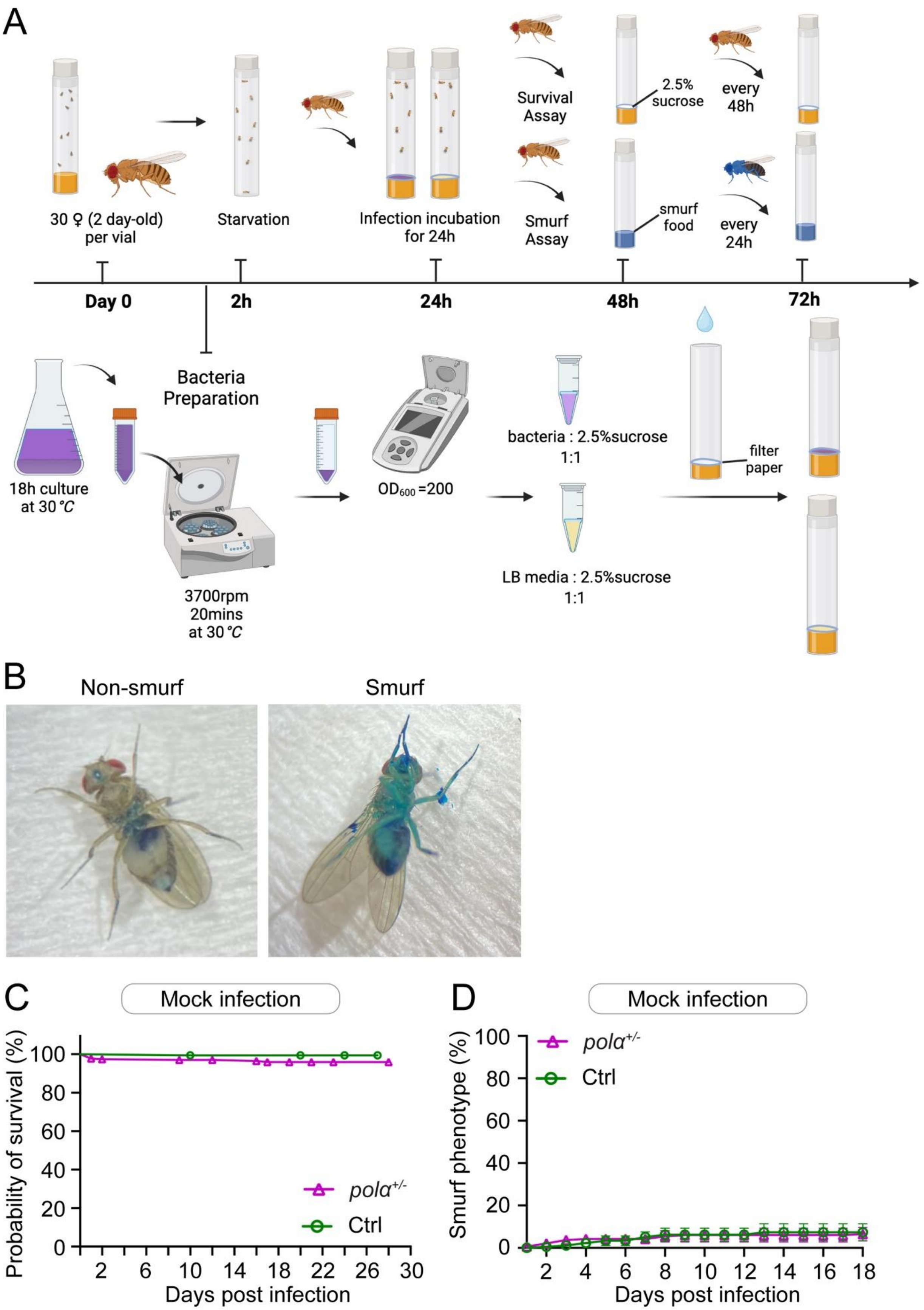

bioRxiv preprint doi: https://doi.org/10.1101/2024.09.19.613993; this version posted September 20, 2024. The copyright holder for this preprint (which was not certified by peer review) is the author/funder, who has granted bioRxiv a license to display the preprint in perpetuity. It is made available under aCC-BY 4.0 International license.

Figure S2: Experimental scheme and control experiments for survival and smurf assays post bacterial infection in *Drosophila* intestine. (**A**) Experimental scheme for bacterial infection inoculation, survival and smurf assays (see Methods and Materials for details). (**B**) Images showing a fly without smurf phenotype and a fly with smurf phenotype. (**C**) Probability of survival for non-infected controls: Flies were fed with a solution of LB media and sucrose as a mock infection for 24 hours, then replaced with fresh food every 48 hours: *pola50*$^{+/-}$ heterozygotes (magenta line, n= 271) *vs.* the control (green line, n = 178), $P > 0.05$ by Kaplan-Meier test: Log-rank (Mantel-Cox) test. (**D**) Smurf assay for non-infected controls: Using the same setting for mock infection as shown in (**C**), flies were flipped onto blue dye solid food daily to monitor the number of flies exhibiting smurf phenotypes: *pola50*$^{+/-}$ heterozygotes (magenta line, n = 209) *vs.* the control (green line, n =178). Two-way ANOVA (mixed-effects), for time factor: P=0.05; for genotype factor: P= 0.98.

bioRxiv preprint doi: https://doi.org/10.1101/2024.09.19.613993; this version posted September 20, 2024. The copyright holder for this preprint (which was not certified by peer review) is the author/funder, who has granted bioRxiv a license to display the preprint in perpetuity. It is made available under aCC-BY 4.0 International license.

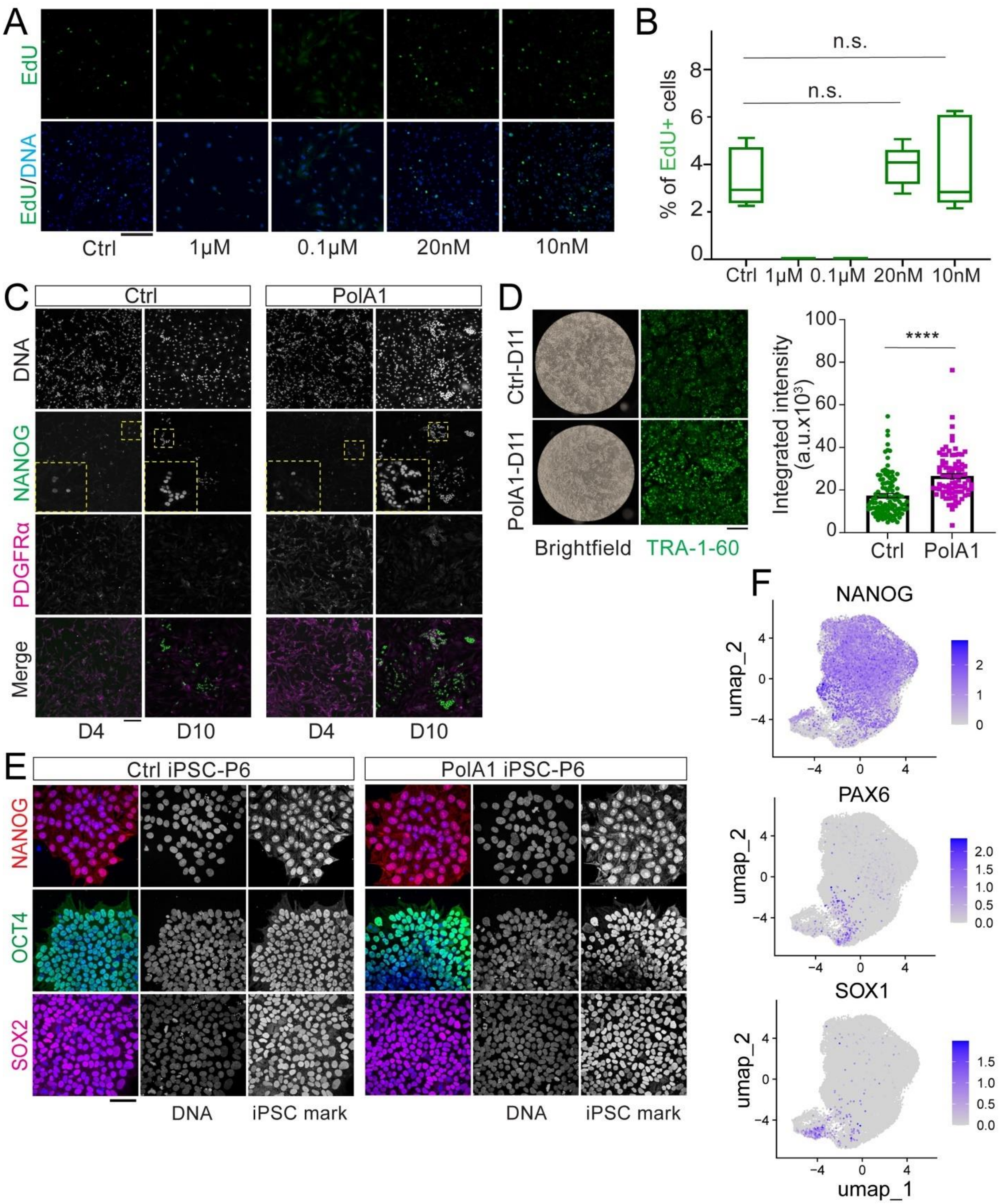

Figure S3: Conditions and resultant cells from PolA1 inhibitor treated human dermal fibroblast cells in iPSC reprogramming process. (**A**) EdU was pulsed for 20 minutes followed

bioRxiv preprint doi: https://doi.org/10.1101/2024.09.19.613993; this version posted September 20, 2024. The copyright holder for this preprint (which was not certified by peer review) is the author/funder, who has granted bioRxiv a license to display the preprint in perpetuity. It is made available under aCC-BY 4.0 International license.

by quantification of EdU+ cells in human dermal fibroblast cells without and with different concentrations of PolA1 inhibitor. (**B**) Compared to the control (4.28±1.56%), no EdU+ cells were detected with PolA1 inhibitor treatment at high concentrations (10 µM, 1 µM, and 0.1 µM). In contrast, low concentrations of PolA1 treatment (0.02 µM: 4.95±1.09%, and 0.01 µM: 4.95±2.45%) show similar percentages of EdU+ cells: P=0.5368 (0.02 µM treatment *vs.* control) and P=0.8413 (0.01 µM treatment *vs.* control). All values = Average ±SEM, statistics was done using Mann Whitney test. (**C**) The NANOG and PDGFRα staining and at D4 and D10 during human iPSC reprogramming. (**D**) The live staining of TRA 1-60 in cells at D11 during reprogramming with and without PolA1 inhibitor. Ctrl: 17424±949.4; PolA1 treated: 26602±1137. ****$P < 10^{-4}$, Average ± SEM by Mann Whitney test. (**E**) Immunofluorescence staining of iPSCs (Passage 6) shows strong expression of pluripotency markers in both control and PolA1 inhibitor treated human iPSCs. The pluripotency markers NANOG, OCT4 and SOX2 were examined using immunostaining. Scale: 50 µm. (**F**) Feature plots show expression of NANOG, a well-known pluripotency stem cell marker in all samples. Two differentiation markers, PAX6 and SOX1, are detected in a very small subset of cells, due to a technical issue with high confluence of the cells (*120*).
